# Pure early zygotic genes in the Asian malaria mosquito *Anopheles stephensi*

**DOI:** 10.1186/s13071-018-3220-y

**Published:** 2018-12-24

**Authors:** Yang Wu, Wanqi Hu, James K. Biedler, Xiao-Guang Chen, Zhijian Jake Tu

**Affiliations:** 1 Department of Pathogen Biology, School of Public Health, Southern Medical, University, Guangzhou, Guangdong 510515 People’s Republic of China; 2 Department of Biochemistry, Engel Hall, Blacksburg, VA 24061 USA; 3 Fralin Life Science Institute, Virginia Tech, Blacksburg, VA 24061 USA

**Keywords:** Embryo, Early zygotic promoter, Development, Gene drive, Vector, Infectious disease

## Abstract

**Background:**

The Asian malaria mosquito, *Anopheles stephensi,* is a major urban malaria vector in the Middle East and on the Indian subcontinent. Early zygotic transcription, which marks the maternal-to-zygotic transition, has not been systematically studied in *An. stephensi* or any other *Anopheles* mosquitoes. Improved understanding of early embryonic gene expression in *An. stephensi* will facilitate genetic and evolutionary studies and help with the development of novel control strategies for this important disease vector.

**Results:**

We obtained RNA-seq data in biological triplicates from four early *An. stephensi* embryonic time points. Using these data, we identified 70 and 153 pure early zygotic genes (pEZGs) under stringent and relaxed conditions, respectively. We show that these pEZGs are enriched in functional groups related to DNA-binding transcription regulators, cell cycle modulators, proteases, transport, and cellular metabolism. On average these pEZGs are shorter and have less introns than other *An. stephensi* genes. Some of the pEZGs may arise *de novo* while others have clear non-pEZG paralogs. There is no or very limited overlap between *An. stephensi* pEZGs and *Drosophila melanogaster* or *Aedes aegypti* pEZGs. Interestingly, the upstream region of *An. stephensi* pEZGs lack significant enrichment of a previously reported TAGteam/VBRGGTA motif found in the regulatory region of pEZGs in *D. melanogaster* and *Ae. aegypti*. However, a GT-rich motif was found in *An. stephensi* pEZGs instead.

**Conclusions:**

We have identified a number of pEZGs whose predicted functions and structures are consistent with their collective roles in the degradation of maternally deposited components, activation of the zygotic genome, cell division, and metabolism. The pEZGs appear to rapidly turn over within the Dipteran order and even within the Culicidae family. These pEZGs, and the shared regulatory motif, could provide the promoter or regulatory sequences to drive gene expression in the syncytial or early cellular blastoderm, a period when the developing embryo is accessible to genetic manipulation. In addition, these molecular resources may be used to achieve sex separation of mosquitoes for sterile insect technique.

**Electronic supplementary material:**

The online version of this article (10.1186/s13071-018-3220-y) contains supplementary material, which is available to authorized users.

## Introduction

The genus *Anopheles* includes dozens of mosquito species that are important vectors of malaria, one of humankind’s most deadly and costly diseases [1]. *Anopheles stephensi*, the Asian malaria mosquito, is a major urban malaria vector in the Middle East and on the Indian subcontinent [2, 3]. Mosquito control contributed significantly to the recent decrease in malaria incidence and mortality [4]. However, insecticide-resistance is widely reported in mosquito populations in malaria-endemic areas of Africa and India [4], leading to considerable interest in developing novel genetic control strategies that specifically target malaria vectors, including *An. stephensi* [5, 6]. In addition, *An. stephensi* is becoming a model for genetic and molecular studies because of the increased availability of genomic resources [7, 8] and genetic manipulation methods including CRISPR/Cas9-mediated genome editing [5].

We are interested in the early embryonic stage when the maternal-to-zygotic transition (MZT) occurs in *An. stephensi*. The MZT includes the syncytial blastoderm and early cellular blastoderm stages, during which the developing embryo is more accessible to genetic manipulation. Thus, the MZT is not only of fundamental importance in embryonic development, it also represents a stage where genetic manipulation could lead to novel mosquito control strategies. In metazoan species, prior to the MZT, the newly formed zygote is transcriptionally inactive and its biological activities are controlled by maternally-deposited RNAs and proteins [9, 10]. During the MZT, transcription of the first set of genes occurs in the nascent zygote while maternally-deposited RNAs and proteins are degraded. In *Drosophila*, the first 13 cycles of nuclear division are rapid and without the formation of new cellular membranes. Thus, in these early embryos, up to thousands of nuclei share the same cytoplasm in a syncytial blastoderm [11]. The minor transcriptional wave is detected as early as cycle 8 when the embryo is still a syncytial blastoderm and the major wave of transcription occurs at or after cell cycle 14 when the cellular blastoderm begins [12]. Many genes expressed prior to the cellular blastoderm stage are essential for sex determination, pattern formation, and cellularization [13]. The precise temporal activation of these genes in the early embryo is critical for normal development thereafter.

We previously identified 61 pure early zygotic genes (pEZGs) in the dengue and yellow fever mosquito *Aedes aegypti* [14]. These pEZGs are defined as genes that have no maternally-deposited transcripts and are transcribed solely during the onset of the MZT. Comparison with the 58 pEZGs that were previously characterized in *D. melanogaster* [15] showed a general lack of overlap of pEZGs between *D. melanogaster* and *Ae. aegypti*. However, the TAGteam and VBRGGTA motifs, two similar regulatory motifs that activate early zygotic transcription were found to be enriched in the upstream sequences of the *Drosophila* and *Aedes* pEZGs [14], respectively. In this study, we obtained RNA-seq data in biological triplicates from four early *An. stephensi* embryonic time points. Using these data, we identified 70 and 153 pEZGs under stringent and relaxed conditions, respectively, and found very limited overlap with either *Drosophila* or *Aedes* pEZGs. A GT-rich motif was found in the *An. stephensi* pEZGs. The TAGteam/VBRGGTA motifs were either not found or found only in a limited number of *An. stephensi* pEZGs. We investigated the structural characteristics of the *An. stephensi* pEZGs and their evolution. We also discuss the potential utility of this study in achieving sex separation of mosquitoes for sterile insect technique.

## Results

### Identification of pure early zygotic genes in *An. stephensi*

**Fig. 1:**
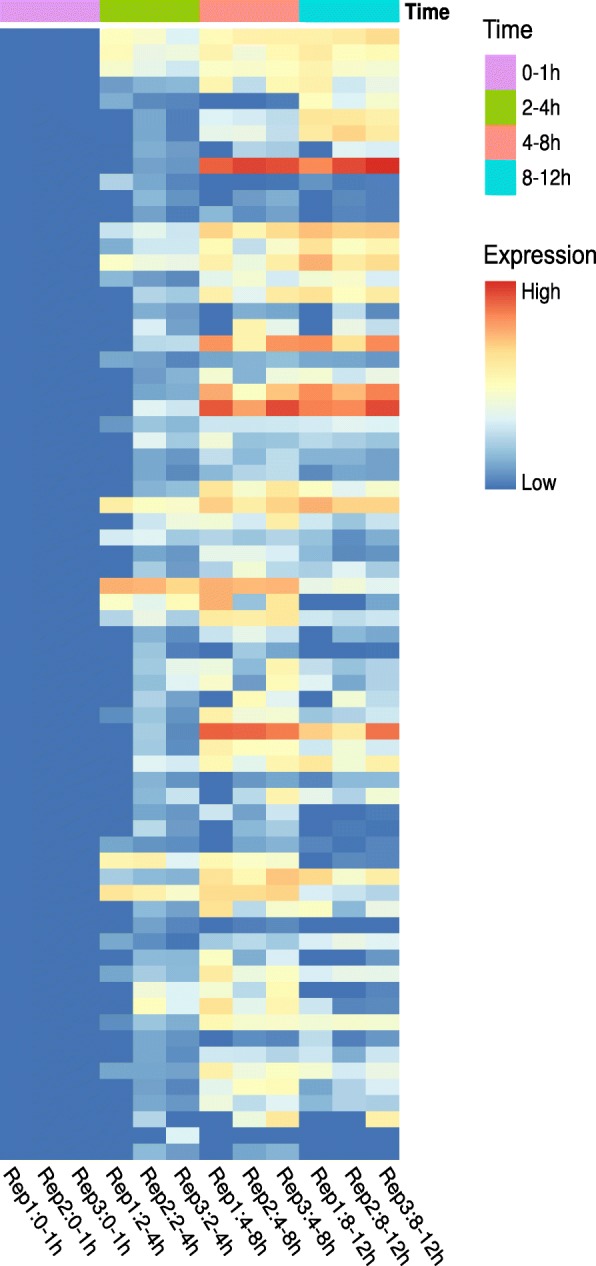
Transcription profile of the 70 *An. stephensi* pure early zygotic genes (pEZGs) spanning early embryonic time points. The FPKM-normalized read counts of each biological replicate were visualized in the heatmap. These 70 genes are identified as pure early zygotic genes using the stringent criteria; thus, as expected, their transcription did not start until the 2-4 h time range

As part of the *An. stephensi* genome project, RNA-seq was performed across different developmental stages, including the embryonic stage [7]. Here, we focused on the period of maternal-to-zygotic transition and obtained RNA-seq data in biological triplicates from early embryos at time points 0-1, 2-4, 4-8, and 8-12 h post-oviposition. These time points were selected because we previously showed that the embryo is not yet transcriptionally active 0-1 h after egg deposition in *An. stephensi* and the syncytial blastoderm stage occurs 3–4 h after egg deposition [16]. To be consistent with previous analyses in *Ae. aegypti* [14] and *D. melanogaster* [15], we focused on the pure early zygotic genes, which are genes that have no maternally deposited transcripts but are initially transcribed during the maternal-to-zygotic transition. Thus, we initially focused on the genes that have no transcript in the 0-1 h pre-zygotic time range but show significant transcript levels during the 2-4 h time range. Seventy genes met the aforementioned criteria as they showed 0 FPKM (fragment per kilobase per million mapped reads) values in the 0-1 h embryos but showed significantly higher expression in the 2-4 h embryos (BH-adjusted *P-*value of 0.05) and had FPKM values of more than 1 in at least one of three 2-4 h embryo replicates. The FPKM value and differential expression analysis of all genes, including the 70 pEZGs, are shown in Additional file 1. Figure 1 shows the early embryonic expression profiles of these 70 pure zygotic genes (pEZGs). The full sequences of these 70 transcripts are shown in Additional file 2.

Similarly, genes showing no transcript in either the 0-1 h or the 2-4 h time ranges but that had significantly higher expression in the 4-8 h time range are defined as pure mid-zygotic genes (pMZGs). Genes that showed transcripts in the 8-12 h time range exclusively are defined as pure late-zygotic genes (pLZGs). In total, 255 and 28 genes were characterized as pMZGs and pLZGs, respectively (Additional file 1). It should be noted that pMZGs and pLZGs are defined relative to the pEZGs. However, all these time points are early in embryonic development as it takes approximately 44 h for the *An. stephensi* embryo to complete development under our rearing conditions.

Potential false positives can result in RNA-seq data when FPKM values are low [17]. Thus, we also applied a relaxed criterion to define the lack of maternally deposited transcript. Under this relaxed criterion, we regarded genes showing less than 1 FPKM in normalized read counts in the 0-1 h embryos as not having maternally-deposited transcripts. In other words, under the relaxed condition, 1 FPKM instead of 0 is used as the cutoff for the lack of maternal deposition in the 0-1 hr embryo. As a result, 153 relaxed pEZGs, including the 70 stringent candidates, were identified (Additional files 1, 2 and 3). We focused our subsequent analysis on the 70 and 153 pEZGs that were identified using the stringent and relaxed criteria, respectively.

### Functional term enrichment and structure of *An. stephensi* pEZGs

**Fig. 2:**
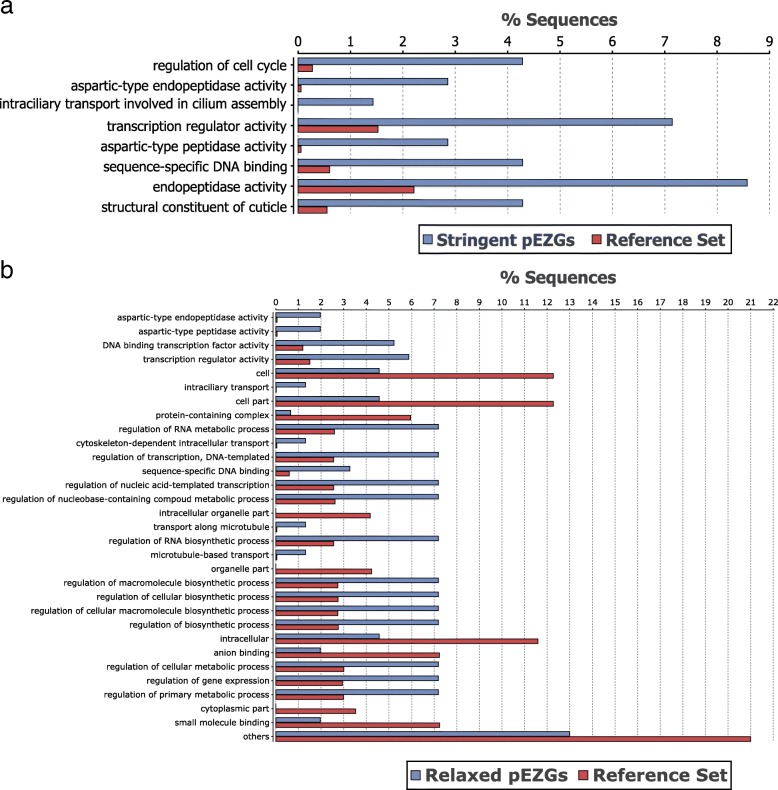
InterPro name enrichment of the *An. stephensi* pure early zygotic genes (pEZGs) identified under stringent (**a**) or relaxed (**b**) conditions. The two sets of pEZGs were analyzed separately against the ASTEI2.2 dataset as the reference. Only names or categories that showed statistically significant (*P* < 0.01) enrichment are shown

*Anopheles stephensi* transcripts (ASTEI2.2) were mapped to the non-redundant database (nr) using BLAST, and the BLAST results were then used to map the *An. stephensi* transcripts to InterPro domain annotations and Gene Ontology (GO) annotations. Two-tailed Fisher's exact tests were conducted for InterPro annotations and GO IDs of the 70 stringent pEZGs and the 153 relaxed pEZGs, respectively, to identify possible enrichments. In both cases, all *An. stephensi* transcripts (ASTEI2.2) were used as the reference for enrichment analysis. Using the *p-*value threshold of 0.01, 8 InterPro names were enriched in the 70 stringent pEZGs (Fig. 2a), and 31 InterPro names were enriched in the 153 relaxed pEZGs (Fig. 2b). In both cases, there is an enrichment in terms of DNA-binding of transcription regulators, cell cycle modulators, proteases, transport, and genes involved in cellular metabolism (Fig. 2). These predicted functions are consistent with cell activities in the earliest maternal-to-zygotic transition stage, where maternal components are degraded and a few transcription factors are expressed in preparation for future major transcription [10]. There are no enriched InterPro names in either the 70 or the 153 pEZGs under a BH adjusted false discovery rate (FDR) of 0.05.

GO term enrichment was also analyzed. For the 70 stringent pEZGs, there was no GO enrichment under an FDR of 0.05, but enrichments were found under a *p* value of 0.01 (Additional file 4: Figure S1A). The enrichment is mainly associated with the mitotic cell cycle, transport, and cellular metabolism. For the 153 relaxed pEZGs, enrichments were found under an FDR of 0.05 (Additional file 4: Figure S1B). The enrichment is also mainly associated with the mitotic cell cycle, transport, and cellular metabolism.

**Fig. 3:**
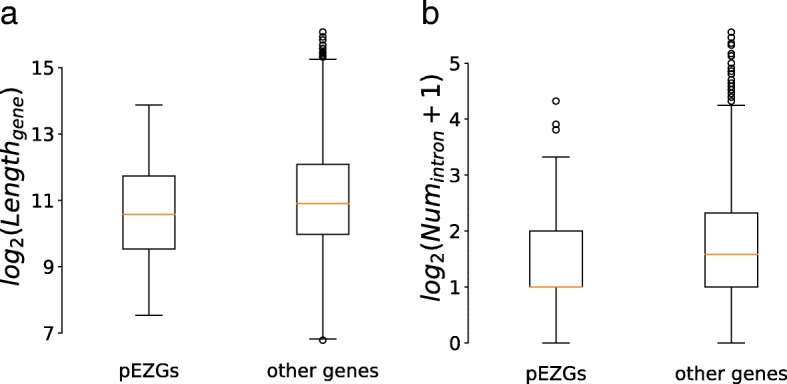
The stringent *An. stephensi* pEZGs are shorter and have fewer introns compared to other *An. stephensi* genes. The length (**a**) and intron number (**b**) of the pEZGs and other *An. stephensi* genes are shown in separate boxplots. Gene length is shown on a log_2_ scale. For better visualization, the number of introns is shown as log_2_ (intron number plus 1). For each box plot, the median is shown as an orange horizontal line, the upper boundary of the box indicates the third quartile (Q3), and the lower boundary of the box indicates the first quartile (Q1). Note that the first quartile of the number of introns in the pEZGs is the same of the median. According to a one-tailed Wilcoxon test, the 70 stringent EZGs are shorter (*P* = 0.0211) and have fewer introns (*P* = 0.005317) compared to other genes in *An. stephensi*

We also investigated the length and intron numbers of the pEZGs. As shown in Fig. 3, the intron number and the median length of the 70 and 153 pEZGs are significantly less than those of other genes (*P* < 0.05), which is consistent with earlier observations in other organisms [15, 18–21].

### Evolution of the *An. stephensi* pEZGs

**Fig. 4:**
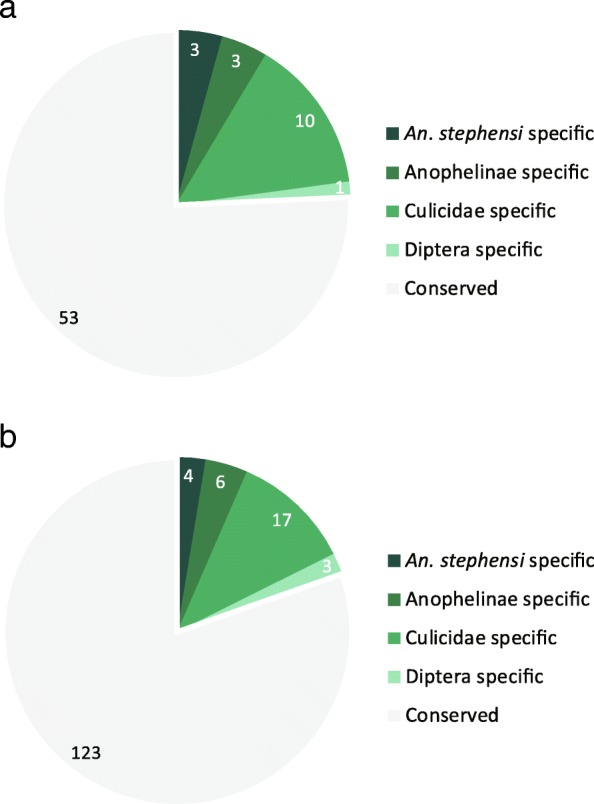
Conserved and lineage-specific pEZGs. The two pie charts show the species distributions of the 70 stringent pEZGs (**a**) and the 153 relaxed pEZGs (**b**). All analyses were done according to OrthoDB comparisons of 12 other insect species as described in the methods section. For example, *An. stephensi*-specific genes refer to genes that are only found in *An. stephensi*, while Anophelinae-specific genes refer to genes that are found in *An. stephensi* as well as other *Anopheles* species surveyed

To investigate how these pEZGs evolved, we constructed phylogenetic trees for each of the 70 stringent pEZGs based on sequence conservation in 13 insect species at the Insecta level (see Methods). Among the 70 *An. stephensi* pEZGs, 17 are only found in Diptera, and 16 of the 17 dipteran genes only exist in mosquitoes, including 6 *Anopheles*-specific genes and 3 genes that are restricted to *An. stephensi*. Among the more inclusive 153 pEZGs, there are 30 Diptera-specific genes, where 27 only exist in mosquitoes, including 10 *Anopheles*-specific genes and 4 genes that are restricted to *An. stephensi* (Fig. 4, Additional file 5: Table S1). Therefore, the *An. stephensi* pEZGs consist of both highly conserved, as well as fast-evolving, lineage-specific genes.

We also investigated the intra-genomic or within-species duplication of the pEZGs. Out of the 70 *An. stephensi* pEZGs, 31 had one or more paralogs in *An. stephensi* (Additional file 6). The transcription profiles of these paralogs revealed that 25 of the 31 multiple-copy genes had at least one paralog expressed either constitutively, or mainly, in non-early zygotic stages at moderate or high levels. Some of the duplications appear to have happened prior to the formation of a common ancestor of mosquitoes (Additional file 6, ASTEI00711 and ASTEI00712). Some other duplications appear to be recent (Additional file 6, ASTEI07878 and ASTEI07879). The remaining 39 pEZGs had no paralog detected, in which 3 were *An. stephensi* specific, suggesting that these three pEZGs were possibly evolved from non­coding regions *de novo*.

### Comparison of pEZGs between An. stephensi, D. melanogaster and Ae. aegypti

**Fig. 5:**
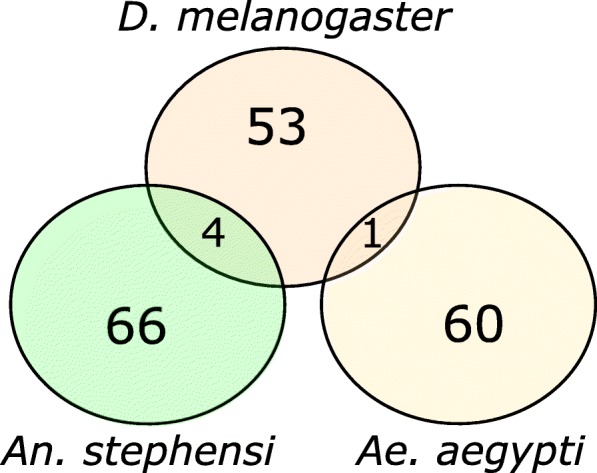
The general lack of shared pEZGs among *An. stephensi*, *D. melanogaster,* and *Ae. aegypti*. The *Drosophila melanogaster* and *Aedes aegypti* pEZGs are from De Renzis *et al.* [15] and Biedler *et al.* [14], respectively. Genes that overlap in the Venn diagram are homologous among the shared species

The 58 and 61 pEZGs that were previously characterized in *D. melanogaster* [15] and *Ae. aegypti* [14] , respectively, were used for comparison with the 70 pEZGs identified here in *An. stephensi*. These pEZGs in all three species were identified under equivalent stringent conditions and represent pEZGs without maternal deposition. The orthologs of *D. melanogaster* and *Ae. aegypti* pEZGs in *An. stephensi* were identified by OrthoDB at the Diptera level. Only one gene (*toll*) and its corresponding ortholog is a pEZG in both *D. melanogaster* and *Ae. aegypti* (Fig. 5). Out of the 70 *An. stephensi* pEZGs, 36 genes had homologous genes in *D. melanogaster;* 16 of them are one-to-one orthologs. There are 4 common genes expressed early and solely zygotically in both *An. stephensi* and *D. melanogaster*, including *tll*, *sna*, *slp1,* and *Bro* (Fig. 5). Twenty-two out of the 70 *An. stephensi* pEZGs had one-to-one orthologs in *Ae. aegypti*. However, no early zygotic gene was shared between these two species (Fig. 5).

### Discovery of a motif potentially involved in early zygotic genome transcription

**Fig. 6:**
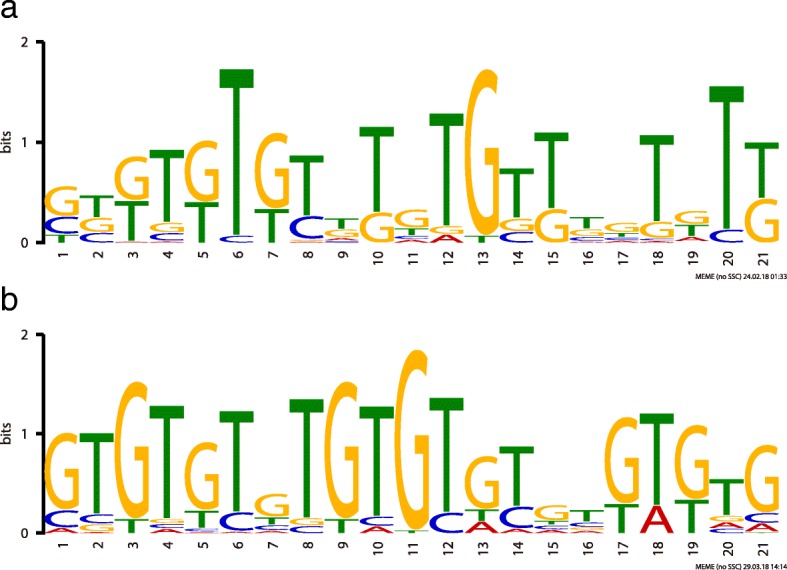
Discovery of motifs that are shared in the putative regulatory regions of the pure early zygotic genes. A significant motif found 1000 bp upstream of the 70 stringent pEZGs (**a**) and 600 bp upstream of the 153 relaxed pEZGs (**b**). Similarity between the two motifs shown in this figure was checked by the STAMP website [24], which concluded that the two motifs are essentially identical with an e-value of 0

To identify potential motifs involved in early zygotic transcription, the upstream sequences relative to the transcription start site (TSS) for each of the 70 stringent pEZGs and the 153 relaxed pEZGs was retrieved from VectorBase BioMart [22] separately and analyzed by the MEME suite [23]. The upstream sequences of all other genes except the 153 relaxed pEZGs were used as a reference or control for each search. For the 70 pEZGs, separate searches were performed using 200, 400, 600, 800 and 1000 bp upstream sequences, with the corresponding upstream sequences of the reference gene set as control. As shown in Fig. 6a, a motif with a low e-value of 6.0e-032 was found in the 1000 bp upstream region in 45 of the 70 stringent pEZGs (Additional file 7). An essentially identical motif was found in all other searches using different upstream sequence lengths with the main difference being the number of motif occurrences found. Similar searches were performed for the upstream sequences of the 153 pEZGs. Due to limitations of the MEME program, only 200, 400 and 600 bp upstream sequences of the 153 pEZGs were analyzed using the control. As shown in Figure 6b, a highly similar GT-rich motif with a low e-value of 3.5e-087 was also discovered in the 600 bp upstream region in 51 of the 153 pEZGs (Additional file 7).

The two GT-rich motifs were nearly identical with an e-value of 0, according to comparison by the STAMP website [24]. Furthermore, the motif shown in Fig. 6a was submitted to GOMo [25], and the motif was associated with several GO terms under a *q* value (BH-adjusted *P-*value) of 0.05. The top 5 predictions included transcription factor activity, protein binding, signal transduction, sequence-specific DNA binding, and axon guidance (Table 1, Additional file 8).

**Table 1:** The top 5 GO terms associated with the GT-rich early zygotic motif

GO term	Score	*P-*value	*q-*value	Specificity	GO name^*^		Gene ID (Rank)
GO:0003700	1.47E-13	1.42E-07	1.02E-05	~83 %	MF	transcription factor activity	FBgn0085375 (2), FBgn0003460 (12), FBgn0042650 (15), FBgn0003300 (32), FBgn0000459 (44), FBgn0085448 (65), FBgn0000964 (89), FBgn0000157 (109), FBgn0004394 (124), FBgn0001185 (151),...390 more...
GO:0005515	1.93E-12	1.42E-07	1.02E-05	~0 %	MF	protein binding	FBgn0085375 (2), FBgn0036713 (6), FBgn0041180 (11), FBgn0003460 (12), FBgn0086782 (17), FBgn0004861 (18), FBgn0003300 (32), FBgn0004860 (54), FBgn0033952 (57), FBgn0037324 (58),...1802 more...
GO:0007165	1.59E-10	1.42E-07	1.02E-05	~8 %	BP	signal transduction	FBgn0036713 (6), FBgn0041180 (11), FBgn0086782 (17), FBgn0015129 (48), FBgn0011589 (55), FBgn0029846 (66), FBgn0000490 (71), FBgn0037976 (79), FBgn0031882 (81), FBgn0015789 (91),...879 more...
GO:0043565	3.30E-09	1.42E-07	1.02E-05	~12 %	MF	sequence-specific DNA binding	FBgn0003460 (12), FBgn0085448 (65), FBgn0000964 (89), FBgn0000157 (109), FBgn0004394 (124), FBgn0003118 (156), FBgn0001320 (170), FBgn0035849 (182), FBgn0003749 (196), FBgn0016076 (212),...223 more...
GO:0007411	5.43E-07	1.42E-07	1.02E-05	~52 %	BP	axon guidance	FBgn0003300 (32), FBgn0259750 (62), FBgn0003138 (76), FBgn0028999 (232), FBgn0015773 (298), FBgn0086680 (312), FBgn0041097 (328), FBgn0001404 (371), FBgn0003328 (404), FBgn0011260 (546),...135 more...

*BP biological process, *CC* cellular component, *MF* molecular function

Two other EZG motifs, the TAGteam motif that activates transcription of the EZGs in *D. melanogaster* and a related VBRGGTA motif that activates transcription of the EZGs in *Ae. aegypti*, were scanned for in the 1 kb upstream sequences of the 70 and 153 *An. stephensi* pEZGs using FIMO [26] in the MEME suite. The TAGteam motif was found in only 14 sites among 153 relaxed pEZG upstream sequences with an average *p* value of 5.35e-05 (Table 2). Only four TAGteam sites were found in the 70 stringent pEZGs (Table 2). No VBRGGTA motif occurrence was found with a *P*-value less than 0.0001.

**Table 2:** The TAGteam motif identified in *An. stephensi* pEZGs

Dataset	Sequence Name	Strand	Start	End	*P-*value	*q-*value^*^	Matched Sequence
Stringent pEZGs (70 genes)	ASTEI01604	-	73	79	5.35e-05	1	CAGGTAG
	ASTEI07457	-	108	114	5.35e-05	1	CAGGTAG
	ASTEI03001	-	160	166	5.35e-05	1	CAGGTAG
	ASTEI08774	-	557	563	5.35e-05	1	CAGGTAG
Relaxed pEZGs (153 genes)	ASTEI05074	+	23	29	5.35e-05	1	CAGGTAG
	ASTEI07271	-	282	288	5.35e-05	1	CAGGTAG
	ASTEI05137	-	378	384	5.35e-05	1	CAGGTAG
	ASTEI03727	+	457	463	5.35e-05	1	CAGGTAG
	ASTEI03869	-	458	464	5.35e-05	1	CAGGTAG
	ASTEI04618	+	514	520	5.35e-05	1	CAGGTAG
	ASTEI08774	-	557	563	5.35e-05	1	CAGGTAG
	ASTEI03727	-	563	569	5.35e-05	1	CAGGTAG
	ASTEI05137	+	790	796	5.35e-05	1	CAGGTAG
	ASTEI03869	-	784	790	5.35e-05	1	CAGGTAG

*Note that the *q* values are poor.

## Discussion

This study represents the first systematic identification of the pEZGs in an *Anopheles* mosquito species. Seventy and 153 pEZGs were identified in *An. stephensi* using stringent and relaxed criteria, respectively. RNA-seq data from carefully staged early embryonic time points in biological triplicates provided the foundation for systematic analysis and statistical power. These pEZGs were enriched in functional groups related to DNA-binding transcription regulators, cell cycle modulators, proteases, transport, and cellular metabolism. This functional enrichment of the pEZGs is consistent with their collective roles in the degradation of maternally deposited components, activation of the zygotic genome, and cellular division and metabolism. Furthermore, these pEZGs were shorter and had less introns than other genes in *An. stephensi*, which is consistent with the theory that rapid nuclear division prior to cellular blastoderm give genes very limited time to be transcribed and/or processed before being interrupted by mitosis [18–21]. Moreover, this study offers new evolutionary insights and potentially useful genomic resources that could facilitate the development of genetic tools for vector control.

### Insights into the evolution and regulation of pEZGs in mosquitoes

Three of the 70 pEZGs identified using stringent criteria and four of the 153 pEZGs identified using relaxed criteria are restricted to *An. stephensi* (Fig. 4). No paralogs were found for these genes, indicating that these pEZGs may have arisen *de novo*. However, many other pEZGs had non-pEZG paralogs. For example, 31 of the 70 *An. stephensi* pEZGs had one or more paralogs in *An. stephensi*. Analysis of their transcription profiles revealed that 25 of the 31 genes had at least one paralog transcribed either constitutively, or mainly in non-early zygotic stage. These results lend further support to the idea that genesis of novel genes through gene duplication followed by functional divergence [27–29].

Another important observation is that the pEZGs appear to rapidly turn over within the family Culicidae. There is no or very limited overlap between *An. stephensi* pEZGs and *Drosophila melanogaster* and *Aedes aegypti* pEZGs (Fig. 5). Thus, this study further extends our previous findings of an overall lack of overlap of pEZGs between two Dipteran species [14] to two species within the family Culicidae. The observed rapid turnover is consistent with the developmental hourglass theory [30], where early and late embryonic developmental stages are thought to show greater divergence. However, it is important to note that many of the genes involved in segmentation and body plan are conserved and expressed in the early embryos of the three species analyzed, although they may not be pEZGs in a particular species because they either have maternally-deposited transcripts or delayed transcription.

In *Drosophila*, the early transcription of pEZGs is controlled by a transcription factor Zelda (zld) and a cis-regulatory element, named the TAGteam motif [13, 31]. Interestingly, the upstream region of the *An. stephensi* pEZGs lacks significant enrichment of either the TAGteam or the VBRGGTA motif, which is found enriched in the regulatory regions of pEZGs in *Ae. aegypti*. However, a GT-rich motif was found in the *An. stephensi* pEZGs instead. It is interesting to note that in *D. melanogaster* there is also a GAGA transcription factor recognizes a regulatory motif in the early zygotic genes, independent of the Zelda-TAGteam system [32].

### Genomic resources to facilitate SIT and other genetic approaches to control mosquito-borne infectious diseases.

*Anopheles stephensi* is a major urban malaria vector in the Middle East and India. This study represents an effort to acquire new genomic resources to improve our understanding of mosquito biology and to facilitate the development of novel strategies to control malaria and other mosquito-borne diseases. We focus on the early embryonic stage when the maternal-to-zygotic transition (MZT) occurs in *An. stephensi*. The MZT includes the syncytial blastoderm and early cellular blastoderm stages, which is when the developing embryo is more accessible to genetic manipulation. Thus, the MZT is not only of fundamental importance in embryonic development, it also represents a stage for genetic manipulation that could lead to novel mosquito control strategies. The pEZGs and the shared regulatory motif reported in this study could provide the promoter or regulatory sequences to drive gene expression in the syncytial or early cellular blastoderm. For example, early embryonic expression of an antidote gene is one of the essential components of a gene drive system that is based on the linkage of a maternally-deposited toxin and a zygotically expressed antidote [33]. Such a gene drive system can be used to drive disease-refractory genes into mosquito populations. An application that is even more relevant to this special issue is that the promoters, or regulatory sequences, provided by these pEZGs could be used to achieve sex-specific lethality, facilitating the establishment of genetic sexing strains [6]. Genetic sexing strains are useful for any genetic methods that are designed to control mosquito-borne infectious disease, as the release of females should be avoided. Genetic sexing strains are especially critical to population suppression approaches, such as sterile insect technique [34–36].

## Conclusions

A number of pEZGs were identified in the Asian malaria mosquito *Anopheles stephensi*. The predicted functions of these pEZGs are consistent with their collective roles in the degradation of maternally deposited components, activation of the zygotic genome, cell division, and metabolism. The pEZGs appear to rapidly turn over within the Dipteran order and within the Culicidae family. These pEZGs, and their shared regulatory motif, could provide the promoter or regulatory sequences to drive gene expression in the syncytial or early cellular blastoderm, a period when the developing embryo is accessible to genetic manipulation. In addition, these molecular resources may be used to achieve sex separation of mosquitoes for sterile insect technique.

## Methods

### Mosquito rearing and RNA sequencing

*Anopheles stephensi* Indian strain was reared at 27 °C at 60 % relative humidity under a 16 h light:8 h dark photoperiod. More than 100 μl of embryos at time points 0-1, 2-4, 4-8 and 8-12 h after egg laying were collected for RNA isolation and subsequent RNA-seq. In total, three biological replicates for each time point were collected for total RNA isolation using TRIZOL RNA isolation reagent (Molecular Research Center), and the quality and quantity of RNA were verified by Bioanalyzer. Library preparation and transcriptome sequencing were performed either by the sequencing facility at Iowa State University or the Biocomplexity Institute at Virginia Tech. The data have been deposited in NCBI with BioProject accession numbers PRJNA168517 and PRJNA451311, respectively.

### Identification of *An. stephensi* EZGs

To establish relative transcript levels in each sample, the RNA-seq reads were mapped to the reference genome of *An. stephensi* Indian strain (ASTIE2.2) using HISAT (v2.1.0) [37]. The genome assembly and annotation were downloaded from VectorBase [22]. The resulting SAM files were sorted using SAMtools (v1.7) [38] and MarkDuplicates from the Picard tool kit (v2.17.10) [39] was applied to identify and remove PCR duplicates. The raw read counting matrix for each gene was generated by an R package, *GenomicAlignments* (v1.14.1) [40], from de-duplicated BAM-formatted files of each sample. Another R package, *DESeq2* (v1.18.1) [41], was used to identify differentially expressed genes between groups. Additionally, the fragments per kilobase per million mapped reads (FPKM) of each gene were calculated by StringTie (v1.3.3b) [42].

RNA-seq data were obtained in biological triplicates from early embryos at time points 0-1, 2-4, 4-8 and 8-12 h after egg laying. These time points were selected because we have previously shown that *An. stephensi* embryos are not yet transcriptionally active during 0-1 h after egg deposition and the syncytial blastoderm stage occurs 3–4 h after egg deposition [16]. Hence, we defined the early zygotic gene (EZG) set as those genes with significantly increased expression (BH-adjusted *P-*value < 0.05) in 2-4 h embryos compared to 0-1 h embryos, and having average FPKMs more than 1 in 2-4 h embryos. To exclude maternal contamination, we applied two different filtering criteria for genes in 0-1 h embryos to obtain two sets of pure early zygotic genes (pEZGs): one is relaxed, in which their FPKMs are no more than 1, and the other is more stringent, in which their FPKMs are required to be equal to 0. Following a similar definition, those genes not expressed until 4-8 h and 8-12 h were identified as pure mid-zygotic genes (pMZGs) and pure late-zygotic genes (pLZGs), respectively. The heat map was generated by *pheatmap* R package (v1.0.8) [43] to illustrate the expression pattern of pEZGs during 0-12 h embryogenesis.

### Function interpretation and enrichment analysis

*Anopheles stephensi* transcripts (ASTEI2.2) were first mapped to the non-redundant database (nr) downloaded from the NCBI ftp server using blastx (v2.7.1) on a high-performance computing system hosted by Virginia Tech. Subsequently, the *An. stephensi* transcripts (ASTEI2.2) were mapped to InterPro domain annotations and GO annotations using Blast2GO Pro (v5.0.13) [44]. The high-performance computer node used was equipped with a 32-core 2 x E5-2683v4 CPU and 128 Gbytes of RAM. Two-tailed Fisher's exact tests were performed for InterPro annotations and GO IDs of *An. stephensi* pEZGs against the entire ASTIE2.2 transcripts to obtain possible enrichments under a *P-*value of 0.01.

### Anopheles stephensi EZG structure

The annotation of the reference genome file in GTF format (ASTEI2.2) was downloaded from VectorBase. An in-house Python script (https://github.com/yangwu91/gene_info, DOI: 10.5281/zenodo.1209380) was designed to count the number of introns and the length for each gene. Then the number of introns and the length were compared for each gene between pEZGs and the rest of the genes. The distributions of intron number and gene length between two datasets were compared separately by the Wilcoxon test.

### Phylogenetic analysis

The homologous genes related to the 70 *An. stephensi* pEZGs found under the stringent criteria in 12 other insects were identified by OrthoDB at the Insecta level [45]. The 12 species database included 5 mosquito species (*Anopheles gambiae*, *Anopheles merus*, *Anopheles sinensis*, *Aedes aegypti* and *Culex quinquefasciatus*), fruit fly (*Drosophila melanogaster*), honey bee (*Apis mellifera*), silkworm (*Bombyx mori*), pea aphid (*Acyrthosiphon pisum*), red flour beetle (*Tribolium castaneum*), human lice (*Pediculus humanus*) and deer tick (*Ixodes scapularis*).

Each *An. stephensi* pEZG was aligned with its homologs in other species using ClustalO (v1.2.4), and poorly aligned regions were trimmed using TrimAl (v1.4) [46] with a “gappyout” option that removes most poorly aligned or poorly represented sequences. The trimmed alignments were then used as input for MrBayes (v3.2.6) [47] to build phylogenetic trees. The parameters used for MrBayes are listed in Additional file 9: Table S2. Trees were arranged and visualized with the *ggtree* R package (v1.11.3) [48].

### Comparison of pEZGs between An. stephensi, D. melanogaster and Ae. aegypti

The 58 and 61 pEZGs that were previously characterized in *D. melanogaster* [15] and *Ae. aegypti* [14], respectively, were used for comparison with the 70 pure early zygotic genes identified in *An. stephensi* under the equivalent stringent condition. They represent pure early zygotic genes without maternal deposition in each of the three species. The orthologs of *D. melanogaster* and *Ae. aegypti* pEZGs in *An. stephensi* were identified by OrthoDB at the Diptera level.

### Bioinformatics identification of an early zygotic motif

The upstream sequences relative to the transcription start site (TSS) for each of the 70 stringent pEZGs and the 153 relaxed pEZGs were retrieved from VectorBase BioMart [22] separately. Because of an incomplete annotation of *An. stephensi* in VectorBase, some retrieved upstream sequences start from the start codon instead of from the TSS. To search for potential motifs, 200, 400, 600, 800 and 1000 bp upstream sequences were used as input for the MEME suite [23] separately. Due to an input data limitation of the MEME suite, only the 200, 400, and 600 bp upstream sequences of the 153 relaxed pEZGs were used as input for the MEME suite [23]. Searches were performed using the discriminative mode, where the corresponding upstream sequences of all *An. stephensi* genes (ASTIE2.2), excluding the 153 relaxed pEZGs, were used as a control for each search for motifs upstream of the 70 stringent pEZGs and 153 relaxed pEZGs. To be inclusive, we used both the default window size (50 nucleotides) and a window of 10 nucleotides. Similarity between candidate motifs was assessed using the STAMP website [24]. Output motifs were also submitted to GOMo [25] to associate possible *D. melanogaster* promoters by estimating the Mann-Whitney rank-sum *P-*value of the GO term's genes, which is an integrated tool in the MEME suite.

Two other EZG motifs, the TAGteam motif that activates *D. melanogaster* early zygotic genome transcription and a related VBRGGTA motif that activates EZG transcription in *Ae. aegypti,* were scanned in the 1 kb upstream sequences of *An. stephensi* pEZGs using FIMO [26] in the MEME suite. Any hits with a *P*-value less than 0.0001 were reported.

## Additional files

Additional file 1: RNA-seq analysis. (XLSX 8372 kb)

Additional file 2: stringent pEZGs. (XLSX 85 kb)

Additional file 3: relaxed (MHT 482 kb)

Additional file 4: **Figure S1.** (XLSX 13 kb)

Additional file 5: **Table S1.** (PDF 1963 kb)

Additional file 6: pEZG paralogs and phylogeny trees. (FASTA 212 kb)

Additional file 7: the GT-rich motif. (XLSX 14 kb)

Additional file 8: GOMo Results. (PDF 635 kb)

Additional file 9: **Table S2.** (FASTA 82 kb)

## References

[1] White NJ, Pukrittayakamee S, Hien TT, Faiz MA, Mokuolu OA, Dondorp AM (2014). Malaria. Lancet..

[2] Rafinejad J, Vatandoost H, Nikpoor F, Abai MR, Shaeghi M, Duchen S (2008). Effect of washing on the bioefficacy of insecticide-treated nets (ITNs) and long-lasting insecticidal nets (LLINs) against main malaria vector *Anopheles stephensi* by three bioassay methods. J Vector Borne Dis.

[3] Gakhar SK, Sharma R, Sharma A (2013). Population genetic structure of malaria vector *Anopheles stephensi* Liston (Diptera: Culicidae). Indian J Exp Biol.

[4] World Health Organization. World malaria report 2017. http://www.who.int/malaria/publications/world-malaria-report-2017/report/en/. Accessed 28 Apr 2018.

[5] Gantz VM, Jasinskiene N, Tatarenkova O, Fazekas A, Macias VM, Bier E (2015). Highly efficient Cas9-mediated gene drive for population modification of the malaria vector mosquito *Anopheles stephensi*. Proc Natl Acad Sci U S A.

[6] Criscione F, Qi Y, Tu Z. GUY1 confers complete female lethality and is a strong candidate for a male-determining factor in *Anopheles stephensi*. Elife. 2016;5. 10.7554/eLife.19281 PMC5061544 27644420

[7] Jiang X, Biedler JK, Qi Y, Hall AB, Tu Z (2015). Complete dosage compensation in *Anopheles stephensi* and the evolution of sex-biased genes in mosquitoes. Genome Biol Evol.

[8] Neafsey DE, Waterhouse RM, Abai MR, Aganezov SS, Alekseyev MA, Allen JE (2015). Mosquito genomics. Highly evolvable malaria vectors: the genomes of 16 *Anopheles* mosquitoes. Science.

[9] Langley AR, Smith JC, Stemple DL, Harvey SA (2014). New insights into the maternal to zygotic transition. Development.

[10] Schier AF (2007). The maternal-zygotic transition: death and birth of RNAs. Science.

[11] Loncar D, Singer SJ (1995). Cell membrane formation during the cellularization of the syncytial blastoderm of *Drosophila*. Proc Natl Acad Sci U S A.

[12] O'Farrell PH, Stumpff J, Su TT (2004). Embryonic cleavage cycles: how is a mouse like a fly?. Curr Biol.

[13] Ten BJ, Benavides JA, Cline TW (2006). The TAGteam DNA motif controls the timing of *Drosophila* pre-blastoderm transcription. Development.

[14] Biedler JK, Hu W, Tae H, Tu Z (2012). Identification of early zygotic genes in the yellow fever mosquito *Aedes aegypti* and discovery of a motif involved in early zygotic genome activation. Plos One.

[15] De Renzis S, Elemento O, Tavazoie S, Wieschaus EF (2007). Unmasking activation of the zygotic genome using chromosomal deletions in the *Drosophila* embryo. Plos Biol.

[16] Criscione F, Qi Y, Saunders R, Hall B, Tu Z (2013). A unique Y gene in the Asian malaria mosquito *Anopheles stephensi* encodes a small lysine-rich protein and is transcribed at the onset of embryonic development. Insect Mol Biol.

[17] Zhang ZH, Jhaveri DJ, Marshall VM, Bauer DC, Edson J, Narayanan RK (2014). A comparative study of techniques for differential expression analysis on RNA-Seq data. Plos One.

[18] Rothe M, Pehl M, Taubert H, Jackle H (1992). Loss of gene function through rapid mitotic cycles in the *Drosophila* embryo. Nature.

[19] McKnight SL, Miller OJ (1976). Ultrastructural patterns of RNA synthesis during early embryogenesis of *Drosophila melanogaster*. Cell.

[20] Shermoen AW, O'Farrell PH (1991). Progression of the cell cycle through mitosis leads to abortion of nascent transcripts. Cell.

[21] Heyn P, Kircher M, Dahl A, Kelso J, Tomancak P, Kalinka AT (2014). The earliest transcribed zygotic genes are short, newly evolved, and different across species. Cell Rep.

[22] Giraldo-Calderon GI, Emrich SJ, MacCallum RM, Maslen G, Dialynas E, Topalis P (2015). VectorBase: an updated bioinformatics resource for invertebrate vectors and other organisms related with human diseases. Nucleic Acids Res.

[23] Bailey TL, Elkan C (1994). Fitting a mixture model by expectation maximization to discover motifs in biopolymers. Proc Int Conf Intell Syst Mol Biol.

[24] Mahony S, Benos PV (2007). STAMP: a web tool for exploring DNA-binding motif similarities. Nucleic Acids Res.

[25] Buske FA, Boden M, Bauer DC, Bailey TL (2010). Assigning roles to DNA regulatory motifs using comparative genomics. Bioinformatics.

[26] Grant CE, Bailey TL, Noble WS (2011). FIMO: scanning for occurrences of a given motif. Bioinformatics.

[27] Ding Y, Zhou Q, Wang W (2012). Origins of new genes and evolution of their novel functions. Annual Review of Ecology, Evolution, and Systematics.

[28] Kaessmann H (2010). Origins, evolution, and phenotypic impact of new genes. Genome Res.

[29] Ponce R, Martinsen L, Vicente LM, Hartl DL. Novel genes from formation to function. International Journal of Evolutionary Biology. 2012;2012. 10.1155/2012/821645 PMC3395120 22811949

[30] Drost HG, Janitza P, Grosse I, Quint M (2017). Cross-kingdom comparison of the developmental hourglass. Curr Opin Genet Dev.

[31] Satija R, Bradley RK (2012). The TAGteam motif facilitates binding of 21 sequence-specific transcription factors in the *Drosophila* embryo. Genome Res.

[32] Blythe SA, Wieschaus EF. Establishment and maintenance of heritable chromatin structure during early *Drosophila* embryogenesis. Elife. 2016;5. 10.7554/eLife.20148 PMC5156528 27879204

[33] Chen CH, Huang H, Ward CM, Su JT, Schaeffer LV, Guo M (2007). A synthetic maternal-effect selfish genetic element drives population replacement in *Drosophila*. Science..

[34] Gilles JR, Schetelig MF, Scolari F, Marec F, Capurro ML, Franz G (2014). Towards mosquito sterile insect technique programmes: exploring genetic, molecular, mechanical and behavioural methods of sex separation in mosquitoes. Acta Trop.

[35] Lees RS, Gilles JR, Hendrichs J, Vreysen MJ, Bourtzis K (2015). Back to the future: the sterile insect technique against mosquito disease vectors. Curr Opin Insect Sci.

[36] Bourtzis K, Lees RS, Hendrichs J, Vreysen MJ (2016). More than one rabbit out of the hat: radiation, transgenic and symbiont-based approaches for sustainable management of mosquito and tsetse fly populations. Acta Trop.

[37] Kim D, Langmead B, Salzberg SL (2015). HISAT: a fast spliced aligner with low memory requirements. Nat Methods.

[38] Li H, Handsaker B, Wysoker A, Fennell T, Ruan J, Homer N (2009). The Sequence Alignment/Map format and SAMtools. Bioinformatics.

[39] Picard Tools. http://broadinstitute.github.io/picard. Accessed 20 March 2018.

[40] Lawrence M, Huber W, Pages H, Aboyoun P, Carlson M, Gentleman R (2013). Software for computing and annotating genomic ranges. Plos Comput Biol.

[41] Love MI, Huber W, Anders S (2014). Moderated estimation of fold change and dispersion for RNA-seq data with DESeq2. Genome Biol.

[42] Pertea M, Pertea GM, Antonescu CM, Chang TC, Mendell JT, Salzberg SL (2015). StringTie enables improved reconstruction of a transcriptome from RNA-seq reads. Nat Biotechnol.

[43] Pheatmap: pretty heatmaps. https://cran.r-project.org/web/packages/pheatmap/index.html. Accessed 20 March 2018.

[44] Gotz S, Garcia-Gomez JM, Terol J, Williams TD, Nagaraj SH, Nueda MJ (2008). High-throughput functional annotation and data mining with the Blast2GO suite. Nucleic Acids Res.

[45] Zdobnov EM, Tegenfeldt F, Kuznetsov D, Waterhouse RM, Simao FA, Ioannidis P (2017). OrthoDB v9.1: cataloging evolutionary and functional annotations for animal, fungal, plant, archaeal, bacterial and viral orthologs. Nucleic Acids Res.

[46] Capella-Gutierrez S, Silla-Martinez JM, Gabaldon T (2009). trimAl: a tool for automated alignment trimming in large-scale phylogenetic analyses. Bioinformatics.

[47] Ronquist F, Teslenko M, van der Mark P, Ayres DL, Darling A, Hohna S (2012). MrBayes 3.2: efficient Bayesian phylogenetic inference and model choice across a large model space. Syst Biol.

[48] Yu G, Smith D, Zhu H, Guan Y, Lam T (2017). ggtree: an R package for visualization and annotation of phylogenetic trees with their covariates and other associated data. Methods Ecol Evol.

