# Supplementary figures and images for: Pure early zygotic genes in the Asian malaria mosquito Anopheles stephensi
Source: Parasit Vectors. 2018 Dec 24;11(Suppl 2):652. doi: 10.1186/s13071-018-3220-y (PMC6304767; doi:10.1186/s13071-018-3220-y)

A

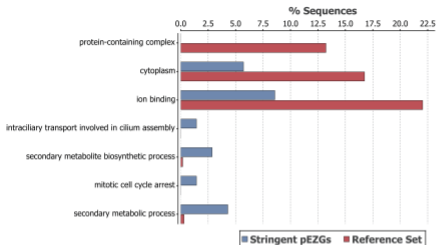

B

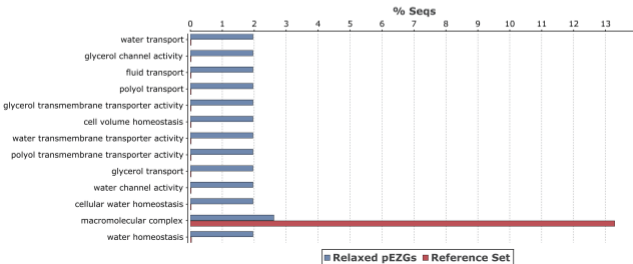

Supplement: Supplementary file 5 — Table S1. (PDF 1963 kb) [file 13071_2018_3220_MOESM5_ESM.pdf]
