# Supplementary material for: Pure early zygotic genes in the Asian malaria mosquito Anopheles stephensi
Source: Parasit Vectors. 2018 Dec 24;11(Suppl 2):652. doi: 10.1186/s13071-018-3220-y (PMC6304767; doi:10.1186/s13071-018-3220-y)
Supplement: Supplementary file 8 — GOMo Results. (PDF 635 kb) [file 13071_2018_3220_MOESM8_ESM.pdf]

Multiple copy genes

pEZGs	corresponding paralogs (pEZGs were colored blue)
ASTEI07879	ASTEI07877,ASTEI07878,ASTEI07879
ASTEI08773	ASTEI09666,ASTEI09669,ASTEI09670,ASTEI09671,ASTEI08774,ASTEI08773
ASTEI02319	ASTEI02322,ASTEI02319
ASTEI11198	ASTEI11198,ASTEI10816,ASTEI11361,ASTEI05893,ASTEI10817
ASTEI08438	ASTEI00533,ASTEI11290,ASTEI09945,ASTEI08438,ASTEI05634,ASTEI08498,ASTEI01696,ASTEI01695,ASTEI09374,ASTEI07099,ASTEI08075,ASTEI08037,ASTEI07100,ASTEI06604,ASTEI06606,ASTEI06607,ASTEI11765,ASTEI10561,ASTEI11381,ASTEI08903,ASTEI08902,ASTEI08439,ASTEI05219
ASTEI01820	ASTEI017980,ASTEI017981,ASTEI11334,ASTEI01819,ASTEI01814,ASTEI01820,ASTEI01816
ASTEI08774	ASTEI09666,ASTEI09669,ASTEI09670,ASTEI09671,ASTEI08774,ASTEI08773
ASTEI05589	ASTEI05590,ASTEI05589
ASTEI00711	ASTEI00711,ASTEI00712
ASTEI01610	ASTEI01609,ASTEI01610
ASTEI11233	ASTEI10928,ASTEI10929,ASTEI10922,ASTEI10927,ASTEI11273,ASTEI11233,ASTEI11234,ASTEI11032,ASTEI11291,ASTEI11525,ASTEI11582,ASTEI11581,ASTEI11422,ASTEI11028
ASTEI06348	ASTEI00668,ASTEI08313,ASTEI06348,ASTEI06939,ASTEI09849,ASTEI01185,ASTEI04816,ASTEI08309,ASTEI063441,ASTEI00271,ASTEI01457,ASTEI08308,ASTEI07582
ASTEI10996	ASTEI01456,ASTEI10996,ASTEI10950
ASTEI00094	ASTEI11462,ASTEI00094,ASTEI03057,ASTEI00850,ASTEI05843,ASTEI11577,ASTEI01072,ASTEI11185,ASTEI09029,ASTEI10311,ASTEI10310,ASTEI02415,ASTEI02414,ASTEI02412
ASTEI07457	ASTEI07456,ASTEI07457
ASTEI05241	ASTEI10556,ASTEI075886,ASTEI04813,ASTEI08921,ASTEI05938,ASTEI08920,ASTEI09253,ASTEI10559,ASTEI10558,ASTEI10557,ASTEI08935,ASTEI00306,ASTEI06383,ASTEI06382,ASTEI05241,ASTEI03461,ASTEI08922,ASTEI09619,ASTEI04800,ASTEI04801,ASTEI10032,ASTEI10033,ASTEI09915,ASTEI02383,ASTEI05106,ASTEI11652,ASTEI05105,ASTEI08923,ASTEI05103,ASTEI05100,ASTEI05101,ASTEI04799,ASTEI10560,ASTEI02516
ASTEI06927	ASTEI06927,ASTEI06928
ASTEI07106	ASTEI07104,ASTEI07106
ASTEI05303	ASTEI11373,ASTEI09803,ASTEI09801,ASTEI00170,ASTEI11571,ASTEI10372,ASTEI11055,ASTEI05303
ASTEI11041	ASTEI11373,ASTEI09803,ASTEI09801,ASTEI00170,ASTEI11571,ASTEI10372,ASTEI11041,ASTEI11055
ASTEI11715	ASTEI10928,ASTEI10929,ASTEI10922,ASTEI10927,ASTEI11273,ASTEI11233,ASTEI11234,ASTEI11032,ASTEI11291,ASTEI11525,ASTEI11715,ASTEI11582,ASTEI11581,ASTEI11422,ASTEI11028
ASTEI01609	ASTEI01609,ASTEI01610
ASTEI11704	ASTEI10167,ASTEI10169,ASTEI09450,ASTEI10170,ASTEI11704
ASTEI07147	ASTEI11196,ASTEI06341,ASTEI01081,ASTEI11006,ASTEI07147,ASTEI11267,ASTEI11200,ASTEI11025,ASTEI10456,ASTEI10457,ASTEI11005,ASTEI11040
ASTEI07148	ASTEI07148,ASTEI11427
ASTEI02027	ASTEI02026,ASTEI05035,ASTEI07316,ASTEI02027
ASTEI10492	ASTEI10492,ASTEI04263,ASTEI08321
ASTEI11635	ASTEI01312,ASTEI01311,ASTEI11635,ASTEI08086,ASTEI10672,ASTEI04709,ASTEI04708,ASTEI085596,ASTEI085597,ASTEI07185,ASTEI08567,ASTEI00489,ASTEI03068
ASTEI04294	ASTEI04293,ASTEI04294
ASTEI11742	ASTEI11463,ASTEI11742
ASTEI06731	ASTEI02593,ASTEI06731

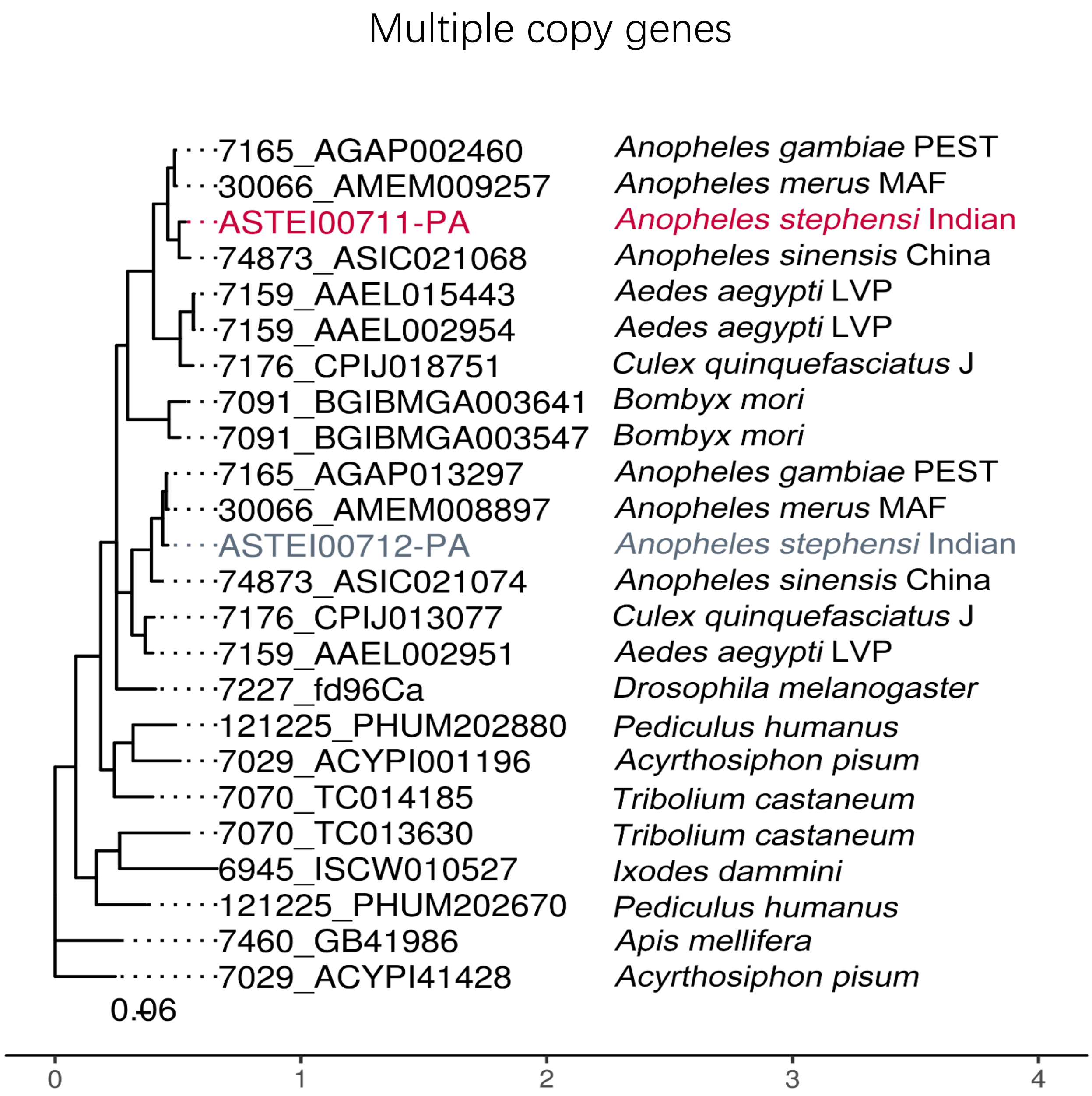

Figure S2. The phylogeny of the *fd96Ca* (forkhead domain 96Ca) gene and its orthologs. *An. stephensi* pEZG (ASTEI00711) was colored red, and its corresponding non-pEZG paralog (ASTEI00712) was colored grey.

Multiple copy genes

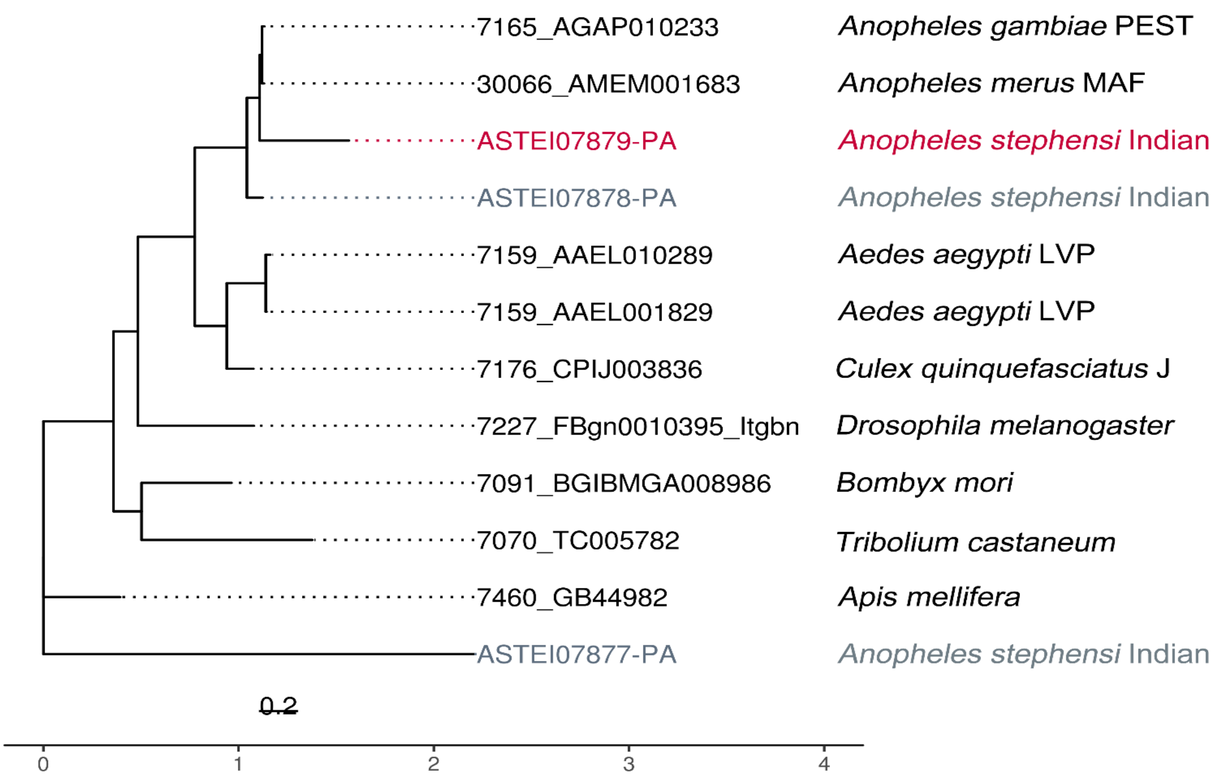

Figure S3. The phylogeny of the *Itgbn* (Integrin beta-nu) gene and its orthologs. *An. stephensi* pEZG (ASTEI07879) was colored red, and its non-pEZG paralogs (ASTEI07877 and ASTEI07878) were in grey. The duplication that led to ASTEI07878 and ASTEI07879 appear to have occurred recently, perhaps after the divergence of the *Anopheles* lineage.
